# Modulation of Brain Activity during a Stroop Inhibitory Task by the Kind of Cognitive Control Required

**DOI:** 10.1371/journal.pone.0041513

**Published:** 2012-07-24

**Authors:** Julien Grandjean, Kevin D’Ostilio, Christophe Phillips, Evelyne Balteau, Christian Degueldre, André Luxen, Pierre Maquet, Eric Salmon, Fabienne Collette

**Affiliations:** 1 Cyclotron Research Center, University of Liège, Liège, Belgium; 2 Department of Psychology: Cognition and Behavior, University of Liège, Liège, Belgium; 3 Department of Electrical Engineering and Computer Science, University of Liège, Liège, Belgium; Charité University Medicine Berlin, Germany

## Abstract

This study used a proportion congruency manipulation in the Stroop task in order to investigate, at the behavioral and brain substrate levels, the predictions derived from the Dual Mechanisms of Control (DMC) account of two distinct modes of cognitive control depending on the task context. Three experimental conditions were created that varied the proportion congruency: mostly incongruent (MI), mostly congruent (MC), and mostly neutral (MN) contexts. A reactive control strategy, which corresponds to transient interference resolution processes after conflict detection, was expected for the rare conflicting stimuli in the MC context, and a proactive strategy, characterized by a sustained task-relevant focus prior to the occurrence of conflict, was expected in the MI context. Results at the behavioral level supported the proactive/reactive distinction, with the replication of the classic proportion congruent effect (i.e., less interference and facilitation effects in the MI context). fMRI data only partially supported our predictions. Whereas reactive control for incongruent trials in the MC context engaged the expected fronto-parietal network including dorsolateral prefrontal cortex (DLPFC) and anterior cingulate cortex, proactive control in the MI context was not associated with any sustained lateral prefrontal cortex activations, contrary to our hypothesis. Surprisingly, incongruent trials in the MI context elicited transient activation in common with incongruent trials in the MC context, especially in DLPFC, superior parietal lobe, and insula. This lack of sustained activity in MI is discussed in reference to the possible involvement of item-specific rather than list-wide mechanisms of control in the implementation of a high task-relevant focus.

## Introduction

Cognitive control serves to adjust and flexibly guide people’s behavior in changing environmental circumstances, especially in situations where distracting information or a prepotent response tendency must be ignored in order to successfully act in a goal-directed manner [1]–[3]. The notion of “cognitive control” can be conceived as a global term that encompasses such well-known concepts in the psychological literature as executive control, goal maintenance, top-down processing, response selection and response inhibition [4].

The Stroop task [5] constitutes one of the most widely used paradigms in cognitive control studies; in this task, an automatic or predominant response tendency (i.e., word reading) must be withheld in favor of a more controlled one [6]. More specifically, subjects are required to name the ink color of color words as fast and accurately as possible. Items in the Stroop task can be congruent, with a match between ink color and color word (e.g., “red” written in red), incongruent (“red” written in green), or neutral (e.g., a non-word written in red). Reaction times (RTs) are typically slower for incongruent than for congruent or neutral trials; this phenomenon is known as the interference effect and is generally considered to reflect the time needed to overcome the conflict between the automatic word-reading tendency and the more controlled color naming response [7], [8]. In addition, a facilitation effect (i.e., faster RTs for congruent than neutral items), also due to inadvertent word reading, has been reported [9], [10].

### The Dual Mechanisms of Control (DMC) Account

Braver et al. [11] developed a general theory of cognitive control, which states that flexibility in cognitive control strategies, depending on situational demands or individual differences, may be achieved through reactive or proactive control [12]. These two processes are clearly separable in terms of cognitive properties and brain activity. Proactive control is a sustained form of control that can be engaged in situations where one can anticipate upcoming stimuli, allowing one to respond rapidly and efficiently by actively maintaining all task-relevant information (e.g., task instructions, identity of previous stimuli, cues for later behavior, etc.). Reactive control, on the other hand, is engaged in situations in which anticipating the upcoming stimuli is not possible, and where the occurrence of a critical event triggers the reactivation of required information in a transient manner. For example, in the context of an interference resolution task such as the Stroop task, reactive control would seek to detect and resolve interference after its onset, whereas proactive control would aim at anticipating and preventing interference before it occurs. Thus, proactive control is not specific to one type of stimulus and reflects a longer period of time of active goal maintenance.

As a result, an important factor that can modulate the extent to which proactive or reactive strategies contribute to task performance is the overall task context (i.e., task demands and characteristics). Indeed, whereas both strategies are equally likely to lead to correct performance on a specific trial, there are some situations in which one or the other kind of control would be most appropriate, with task context encouraging the adoption of one form of control over the other. For example, the proportion congruent effect noted in the Stroop literature [13]–[15] reflects how task context can influence performance. Classically, the proportion congruent effect is the observation of less interference and facilitation in lists of stimuli containing mainly incongruent items (low-proportion-congruent condition) than in lists containing mainly congruent trials (high-proportion-congruent condition; [16], [17]).

Expectations are seen as playing a crucial role in the phenomenon [15], [18], and are used by participants as a cue to adjust the influence of word-reading processes on performance. In high-proportion-congruent situations, the need to rely exclusively on color naming processes is not perceived as crucial given that a majority of trials can be successfully responded to simply by reading the words. On the contrary, in low-proportion-congruent situations, color naming would be the main determinant of response for all the trials within the list, given the high probability of errors associated with word reading processes. Consequently, the interference associated with incongruent trials is less pronounced, but this is also true for the facilitation effect, given that the same processing is applied to all the items. Importantly, this proportion congruent effect can be explained within the DMC account and the proactive/reactive control distinction [11]. More specifically, the low-proportion-congruent condition would be associated with a proactive control strategy, with sustained high activation of goal-relevant information (inhibiting word-reading processes in favor of color naming), whereas the high-proportion-congruent condition would be associated with the reactive control strategy, with transient recruitment of attentional control for critical interfering items only.

Importantly, both mechanisms of control are claimed to be clearly dissociable at the brain level. Specifically, one of the key hypotheses within the DMC account is that proactive and reactive control mechanisms also differ according to the brain regions subserving them and the temporal pattern of neural activity. Along these lines, interactions between the lateral prefrontal cortex (PFC) and anterior cingulate cortex (ACC) constitute a core characteristic of how cognitive control is implemented within the DMC model during an interference task [11], [12], [19]. More specifically, reactive control is assumed to be associated with transient activation of the lateral PFC when interference is detected (reactivation of task goals), and proactive control with sustained activation of the lateral PFC (active maintenance of task goals). A wider network of additional brain regions typically associated with conflict detection and monitoring, especially the ACC, is also expected to play a crucial role for reactive control in Braver et al’s account.

The Stroop task has consistently been associated with a large fronto-parietal network, typically involving the ACC, dorsolateral prefrontal cortex (DLPFC), inferior frontal gyrus, inferior and superior parietal cortex and insula [20]–[22]. Moreover, a series of studies seems to indicate, as suggested by Braver and colleagues [11], [19], that the ACC and DLPFC play differential roles in conflict resolution [23]. In addition, greater involvement by the ACC has been observed for incongruent trials in lists containing few incongruent trials than in lists composed mostly of incongruent trials [2], [24]. The ACC-DLPFC network is also differentially involved after probabilistic cueing, with greater conflict effects in the ACC and DLPFC for incongruent items following highly congruent-predictive cues by comparison to incongruent items following highly incongruent-predictive or non-predictive cues [25]. Finally, Kerns et al. [2] showed that the activity in the ACC for conflicting trials predicted subsequent PFC activity and adjustments in behavior. In this context, Botvinick et al. [1], [26], in their conflict monitoring hypothesis, proposed that the ACC is involved in conflict detection and monitoring and will recruit the DLPFC when interference occurs in order to resolve conflict in a top-down manner by means of strategic adjustments in cognitive control. In order to select the appropriate response, the DLPFC would bias information processing in posterior brain regions (i.e., parietal cortex) to favor the most relevant criteria for performing the task.

### Neuroimaging Studies of Proactive and Reactive Control

Surprisingly, the DMC account has received little attention in neuroimaging studies of the Stroop task, and most of the studies used other cognitive control paradigms, such as the AX version of the Continuous Performance Task (AX-CPT), a task considered to evaluate goal representation, maintenance, and updating. In this task, participants are presented with cue-probe pairs, and must make a target response to an X-probe only when preceded by an A-cue. Non-target responses must be given for all other trials (“BX”, “AY”, and “BY” trials). Hence, contextual cues serve as task goal-relevant information regarding the correct response to produce following ambiguous probes (for a further description of this task, see [27]). More specifically, in a series of publication, Braver and colleagues obtained behavioral and brain data indicating flexible involvement of proactive and reactive control depending on task context [28]–[31]. For example, Locke and Braver [28] showed a shift from reactive to proactive control (i.e. active maintenance of cue letter during the delay and increased sustained activity in a network including the right lateral PFC) during reward incentive task blocks in comparison to baseline or penalty blocks. In a further study, Braver et al. [29] showed a shift from an anticipatory, sustained control (cue-related pattern of activity in lateral PFC that would represent active maintenance of goal-relevant information during the cue-probe delay) to a just-in-time control engaged during task probe occurrence during penalty blocks. In addition, Paxton et al. [32] also provided neuroimaging evidence showing a shift from proactive to reactive control with advancing age. Indeed, they demonstrated an age-related shift in lateral PFC regions, with reduced cue-related activity and increased probe-related activity for older than for younger participants (see also [33], for similar data obtained with a task-switching paradigm). However, following a period of task-strategy training, older may shift to a proactive strategy [29].

Regarding the Stroop task, Carter et al. [24], as indicated previously, showed greater involvement of the ACC for incongruent trials in mostly congruent situations than in mostly incongruent situations. More recently, Floden et al. [34] explored neural substrates associated with the Stroop effect according to task context. Three task context manipulations were used: a blocked context (all trial types were identical within a run), an unblocked-uncued context (all trials were intermixed and completely unpredictable), and an unblocked-cued context (intermixed trials each preceded by a cue signaling the upcoming trial type). The results showed transient ACC and DLPFC activation mainly for incongruent trials in the unblocked and uncued condition, which supports the hypothesis that these areas are involved in reactive control processes.

The studies by Carter et al. [24] and Floden et al. [34] were not expressly designed to evaluate the complete pattern of brain areas associated with the respective contributions of reactive and proactive control mechanisms. Indeed, although Carter et al. focused on the ACC and aimed at better understanding its role in conflict resolution (i.e., conflict detection vs. strategic process implementation), Floden et al. did not consider the proactive/reactive control distinction at all, and aimed at comparing blocked versus unblocked task contexts rather than low- versus high-proportion-congruent conditions. Therefore, the present study investigated whether the general task context (i.e., the proportion congruency) may influence the mode of cognitive control that drives performance.

At the behavioral level, we should observe less interference from incongruent and less facilitation from congruent trials in the mostly incongruent than in the mostly congruent condition (proactive control). On the contrary, the greater reliance on word reading in a mostly congruent context should favor the occurrence of greater interference and facilitation effects, with reactive control (reactivation of task goals) occurring only for incongruent stimuli. At the brain level, we expected incongruent trials of the mostly congruent conditions to elicit a transient activation of the ACC and lateral PFC, reflecting reactivation of task goals and interference resolution (reactive control). On the contrary, we did not expect any transient activation in the mostly incongruent context. Rather, we expected a sustained activation (across trials) in the lateral PFC during this context, reflecting active goal maintenance for all the items presented (proactive control).

## Materials and Methods

### Ethics Statement

The study was approved by the Ethics Committee of the Faculty of Medicine of the University of Liège. In accordance with the Declaration of Helsinki, all participants gave their written informed consent prior to their inclusion in the study.

### Participants

Twenty-eight right-handed native French-speaking young adults, with no diagnosed psychological or neurological disorders, were recruited from the university community. All had normal color vision. Each participant was also screened for any physical or medical condition that could prevent an MRI session. Three participants were excluded from analysis because of incomplete data or technical problems during scanning that precluded their inclusion in further analyses. The 25 participants who remained for the statistical analyses included 12 men and 13 women. Their ages ranged from 18 to 29 years (mean = 21.8±2.68).

### Materials

**Figure 1:**
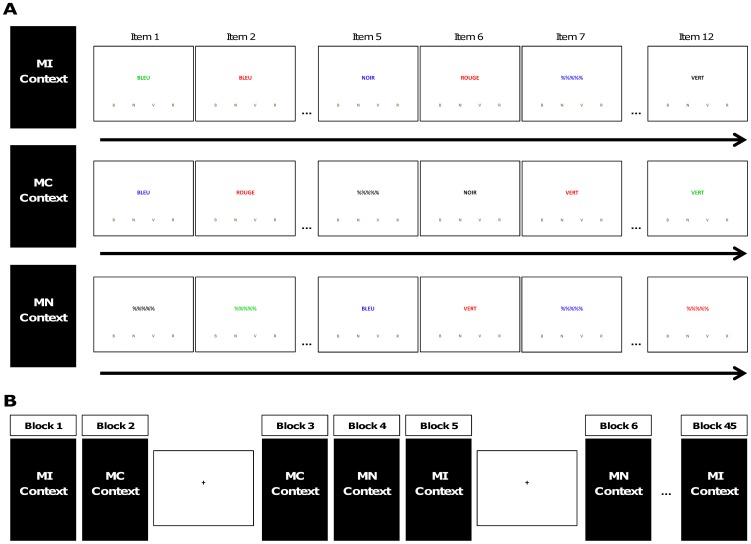
Proportion congruency manipulation. (a) Presentation of the three task contexts (MI, MC, and MN) used in this experiment, with twelve items constituting each MI (8 incongruent, 2 congruent, and 2 neutral items), MC (2 incongruent, 8 congruent, and 2 neutral items), and MN (2 incongruent, 2 congruent, and 8 neutral items) block, and (b) general procedure for context presentation with a fixation cross presented after every two or three blocks of stimuli, for a total of 45 blocks.

Four color words presented on a white background were used in this experiment (Red, Blue, Black, and Green). Proportion congruency was manipulated using three different contexts of 12 items each (see Figure 1a): the *mostly congruent context* (MC), the *mostly incongruent context* (MI), and the *mostly neutral context* (MN). Each MI block was composed of 8 incongruent items (e.g. the word “red” written in “blue”), 2 congruent items (e.g. the word “blue” in “blue”), and 2 neutral items, which were non-verbal stimuli (i.e., strings of five percent signs %%%%%) presented in one of the four color possibilities. For the MC context, the proportions of congruent and incongruent items were reversed. Finally, 8 neutral, 2 congruent, and 2 incongruent items were presented during the MN context. Importantly, the first four items in each block were representative of the current task context (e.g., four incongruent trials in the beginning of each MI context) and served to induce the use of proactive or reactive control processes. The presentation order of the different blocks was pseudo-randomized, with the use of three different presentation orders. Each of the three congruency conditions of 12 items (MI, MC, and MN contexts) was presented 15 times, for a total of 45 blocks and 540 items.

### Procedure

Participants were instructed that their task would be to select the color in which each item was printed. They were told that the items would be presented briefly and they were to respond as fast and accurately as possible. Color words were presented on a screen that the participants viewed through a mirror located on the scanner’s head coil. Each trial consisted of the presentation of a word in the center of the screen, with four response possibilities (written in brown, a color never used for the items) at the bottom of the screen (Figure 1a). Participants had thus to press one of the four response keys on a keyboard, which corresponded to the four color ink possibilities, always in the same order (blue, black, green, red, respectively). They used the index and the middle fingers of their left and right hands for responding. Each item was presented until the participant responded (with a maximum presentation time of 2000 ms). If the participant responded before the deadline, a white screen was presented for the remaining period. If no response was provided, a white screen appeared after 2000 ms and an inter-stimulus interval of 500 ms occurred before the next item. A fixation cross was presented in the center of the screen for 5000 milliseconds after every two or three contexts to provide breaks during the experiment (Figure 1b).

Prior to the MRI session, participants performed a practice session outside the scanner in which 40 items were presented in order to be sure that they understood the task instructions. In the fMRI scanner, four more examples were presented just before the test phase began. After the session, participants received a debriefing that explained the main objective of the experiment.

### Behavioral Data Analysis

All behavioral data met the criteria of normal distribution and the sphericity assumption, and were analyzed with a statistical level set at *p*<.05. Repeated measures ANOVAs were run on the mean RTs and accuracy data (errors and no response), with task context (MC, MI, and MN context) and item type (incongruent, congruent, and neutral) as repeated measures factors. We also reported partial eta squared (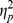) as a measure of effect size. Finally, post hoc comparisons were performed, also with a *p*<.05, using pairwise Tukey’s tests.

### MRI Acquisition

Functional MRI time series were acquired on a 3T head-only scanner (Magnetom Allegra, Siemens Medical Solutions, Erlangen, Germany) operated with the standard transmit-receive quadrature head coil. Multislice T2*-weighted functional images were acquired with a gradient-echo echo-planar imaging sequence using axial slice orientation and covering the whole brain (32 slices, FoV = 220×220 mm^2^, voxel size 3.4×3.4×3 mm^3^, 30% interslice gap, matrix size 64×64×32, TR = 2130 ms, TE = 40 ms, FA = 90°). For anatomical reference, a high-resolution T1-weighted image (3D MDEFT) was acquired for each subject [35] (TR = 7.92 ms, TE = 2.4 ms, TI = 910 ms, FA = 15°, FoV = 256×224×176 mm^3^, 1 mm isotropic spatial resolution). The first three volumes were discarded to avoid T1 saturation effects. Head movement was minimized by restraining the subject’s head using a vacuum cushion. Stimuli were displayed on a screen positioned at the rear of the scanner, which the participant could comfortably see through a mirror mounted on the standard head coil.

### fMRI Data Analyses

Data were preprocessed and analyzed using SPM8 (Wellcome Trust Centre for Neuroimaging, http://www.fil.ion.ucl.ac.uk/spm) implemented in MATLAB 7.5.0 (Mathworks Inc., Sherborn, MA). Images of each individual participant were first realigned (motion corrected). After this realignment, we spatially coregistered the mean EPI image to the anatomical MRI image and coregistration parameters were applied to the realigned BOLD time series. Individual anatomical MRIs were spatially normalized into the MNI space (Montreal Neurological Institute, http://www.bic.mni.mcgill.ca), and the normalization parameters were subsequently applied to the individually coregistered BOLD times series, which was then smoothed using an isotropic 8-mm full-width at half-maximum (FWHM) Gaussian kernel.

**Figure 2:**
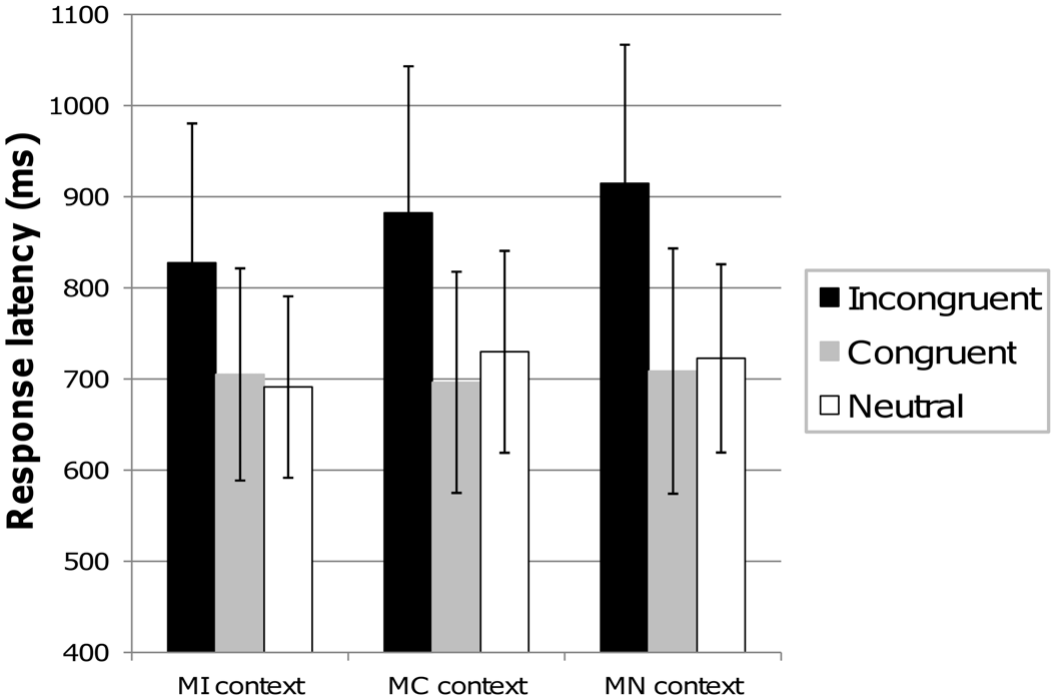
Behavioral results. Mean reaction times (ms) in the MI, MC, and MN contexts for incongruent, congruent and neutral items. An interference effect (incongruent vs. neutral) was observed in each context, and incongruent trials were responded faster in the MI than in the MC and MN contexts. A facilitation effect (congruent vs. neutral) was only observed in the MC context. Error bars represent standard deviations.

**Table 1:** Accuracy data (percentage of errors and no responses) in the MI, MC, and MN contexts for incongruent, congruent and neutral items.

	MI context	MC context	MN context
Incongruent	5.21 (2.88)	6.53 (5.60)	5.14 (5.11)
Congruent	1.67 (2.78)	1.88 (2.66)	1.53 (2.60)
Neutral	1.11 (2.12)	3.89 (4.78)	2.78 (2.72)

*Note*: Numbers in parentheses correspond to standard deviations.

For each participant, BOLD responses were modeled at each voxel, using a general linear model with events convolved with the canonical hemodynamic response function as regressors. Events were divided according to the three contexts (MI, MC, or MN context) and the type of item (incongruent, congruent, or neutral). These 9 regressors were modeled as event-related responses. Event durations corresponded to the presentation of the item until the subject’s response, with a maximum duration of 2 s. Incorrect trials and no responses were also modeled as separate regressors. The design matrix also included the realignment parameters to account for any residual movement-related effect. In addition, the first four items for each context were modeled separately in the design matrix. The rationale for excluding these items was that they did not fully reflect the cognitive control strategy postulated for the context in question (i.e., in the MI context, the first items served to establish the subsequent proactive control strategy by creating expectations associated with this context, and similarly in the MC context, the first items created a low expectation of incongruent trials). A high pass filter was implemented using a cut-off period of 256 s in order to remove the low-frequency drifts from the time series. Linear contrasts assessed the simple main effect of each trial type. The resulting set of voxel values constituted a map of *t* statistics, SPM[T]. The corresponding contrast images were smoothed (6-mm FWHM Gaussian kernel) and entered into a second-level analysis, corresponding to a random-effect model. All analyses were conducted using a correction for multiple comparisons at the voxel level with a conservative family-wise error (FWE) threshold *p*<.05.

**Table 2:** General interference effect (incongruent vs. neutral in MI, MC, and MN context).

Hemisphere	Anatomical region	MNI coordinates			Z score	P value
		x	y	z		
L	Inferior frontal	−42	14	26	6.99	**<.001**
L	Inferior frontal	−54	20	34	6.77	**<.001**
L	Middle frontal	−44	38	34	4.77	**.013**
L	Superior frontal	−24	4	68	5.12	**.003**
L	Anterior cingulate	−2	18	50	4.60	**.027**
L	Anterior insula	−34	20	2	6.12	**<.001**
R	Anterior insula	32	22	4	5.22	**.002**
R	Inferior frontal	30	24	−10	4.51	**.039**
L	Superior parietal	−24	−70	42	6.87	**<.001**
L	Inferior parietal	−32	−50	48	5.96	**<.001**
L	Precuneus	−8	−64	62	5.68	**<.001**
R	Intraparietal sulcus	30	−50	44	4.78	**.013**
L	Inferior occipital	−44	−86	−8	6.28	**<.001**
L	Fusiform gyrus	−48	−56	−20	6.20	**<.001**
L	Inferior occipital	−42	−68	−10	5.83	**<.001**
L	Superior temporal	−54	−46	14	5.19	**.002**
R	Cerebellum	30	−64	−34	5.15	**.002**
R	Cerebellum	10	−76	−28	5.14	**.003**
R	Cerebellum	46	−52	−42	4.93	**.007**

L/R = left or right; x, y, z: coordinates (mm) in the stereotactic space defined by the Montreal Neurological Institute (MNI). This analysis was conducted with a *p* value <.05 FWE corrected.

A 3 (context)×3 (item type) whole-brain voxel-wise repeated measures ANOVA was performed, which allowed us to examine the brain regions related to the comparisons of interest (i.e., general interference effect in the three contexts, interference effects in each context separately, comparison of incongruent trials in the MI and MC contexts with neutral trials in the MN context, comparison of brain activity across the MI context vs. across MC or MN contexts). To further investigate similarities and differences in the activation maps between incongruent trials in the MI and MC contexts, supplementary conjunction and interaction analyses were performed between the interference effects in the MC and MI contexts (respectively assessed here by the comparison between incongruent and neutral trials in the MC context, and the comparison between incongruent trials in the MI context and neutral trials in the MN context). More specifically, the conjunction analysis using the null hypothesis [36] aimed at investigating common brain activations in both contrasts. Given the conservative nature of this conjunction analysis, all activations with a *p* value <.001 uncorrected were reported [37], [38]. The interaction analysis aimed at investigating the specific pattern of activation related to the interference effect in the MC context. As with the conjunction analysis, we reported all activations with a *p* value <.001 uncorrected.

**Figure 3:**
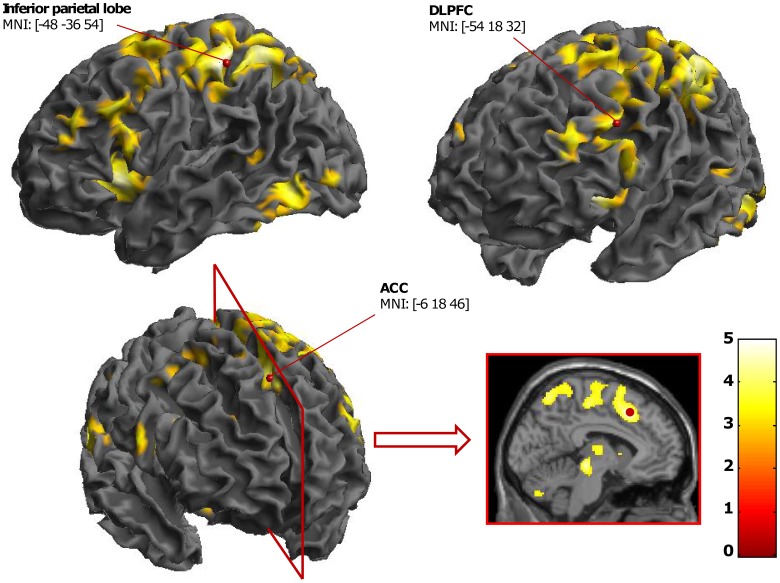
fMRI results for the interference effect in the MC context. This contrast evidenced ACC, DLPFC, and inferior parietal lobe activations (statistical threshold at *p*<.001 uncorrected for the present display).

**Table 3:** Interference effect (incongruent vs. neutral items) in MI, MC and MN contexts.

Hemisphere	Anatomical region	MNI coordinates			Z score	P value
		x	y	z		
**a) Interference effect in MC context**						
L	Inferior parietal	−48	−36	54	4.82	**.011**
L	Inferior parietal	−32	−52	50	4.56	**.033**
L	Superior parietal	−32	−54	66	4.71	**.017**
L	Superior parietal	−26	−68	38	4.61	**.027**
L	Middle frontal	−26	−10	54	4.77	**.014**
L	Inferior frontal	−52	16	28	4.18	.001 a
L	Anterior cingulate	−6	18	46	4.66	**.021**
L	Anterior insula	−34	14	−6	4.68	**.020**
R	Caudate nucleus	14	10	6	4.65	**.023**
L	Inferior occipital	−48	−68	−6	4.63	**.024**
**b) Interference effect in MI context**						
Nil						
**c) Interference effect in MN context**						
L	Inferior frontal	−44	14	28	5.76	**<.001**
L	Inferior frontal	−56	20	34	5.37	**.001**
L	Anterior insula	−32	22	0	4.75	**.015**
L	Middle frontal	−26	46	16	4.53	**.037**
R	Inferior frontal	60	6	32	4.77	**.014**
L	Superior parietal	−24	−70	42	5.45	**.001**
L	Inferior occipital	−42	−88	−10	5.60	**<.001**
L	Inferior occipital	−40	−70	−12	5.48	**<.001**
L	Fusiform gyrus	−50	−58	−20	5.48	**<.001**
R	Inferior occipital	44	−84	−10	4.51	**.040**
R	Cerebellum	8	−76	−30	4.75	**.015**

L/R = left or right; x, y, z: coordinates (mm) in the stereotactic space defined by the Montreal Neurological Institute (MNI). This analysis was conducted with a *p* value <.05 FWE corrected.

a P<.05 FWE corrected with SVC using a 10-mm sphere radius centered on the DLPFC’s MNI coordinates [−48 15 20] [22].

## Results

### Behavioral Data

A 3 context (MI, MC, MN)×3 item type (incongruent, congruent, neutral) repeated measures ANOVA was conducted on the mean RTs (see Figure 2), and revealed a main effect of context (*F*(2,48) = 11.44; *p*<.0001;  = .32), showing faster RTs in the MI context; a main effect of item (*F*(2,48) = 126.81; *p*<.0001; 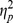 = .84), showing longer RTs for incongruent trials; and a significant context × item interaction (*F*(4,96) = 11.05; *p*<.0001; 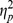 = .32). The analysis of the interaction effect (post hoc Tukey’s tests) showed an interference effect in all three contexts (all *p*s <.001). However, RTs for incongruent trials were faster for the MI context than the MC and MN contexts (all *p*s <.001), and faster for the MC than the MN context (*p* = .04). A facilitation effect (faster RTs for congruent than neutral items) was present only in the MC context, where congruent trials are very frequent (*p* = .03). Finally, reaction times for congruent items did not differ between MI and MC contexts (*p*>.05).

**Table 4:** Comparison of incongruent trials in the MC and MI contexts with neutral trials in the MN context.

Hemisphere	Anatomical region	MNI coordinates			Z score	P value
		x	y	z		
**a) Incongruent trials in MC context versus neutral trials in MN context**						
L	Superior parietal	−26	−68	38	5.23	**.002**
L	Inferior parietal	−50	−36	50	5.20	**.002**
L	Inferior parietal	−36	−40	40	4.48	**.044**
L	Anterior insula	−32	20	−2	5.76	**<.001**
L	Inferior frontal	−48	14	28	5.62	**<.001**
L	Anterior cingulate	−6	20	44	5.16	**.002**
R	Anterior insula	34	20	4	4.99	**.005**
R	Inferior frontal	32	24	−10	4.72	**.017**
R	Caudate nucleus	16	10	8	4.72	**.017**
L	Inferior occipital	−40	−88	−6	4.69	**.019**
R	Superior temporal	68	−42	16	4.61	**.027**
**b) Incongruent trials in MI context versus neutral trials in MN context**						
L	Inferior frontal	−42	14	26	5.06	**.004**

L/R = left or right; x, y, z: coordinates (mm) in the stereotactic space defined by the Montreal Neurological Institute (MNI). This analysis was conducted with a *p* value <.05 FWE corrected.

**Figure 4:**
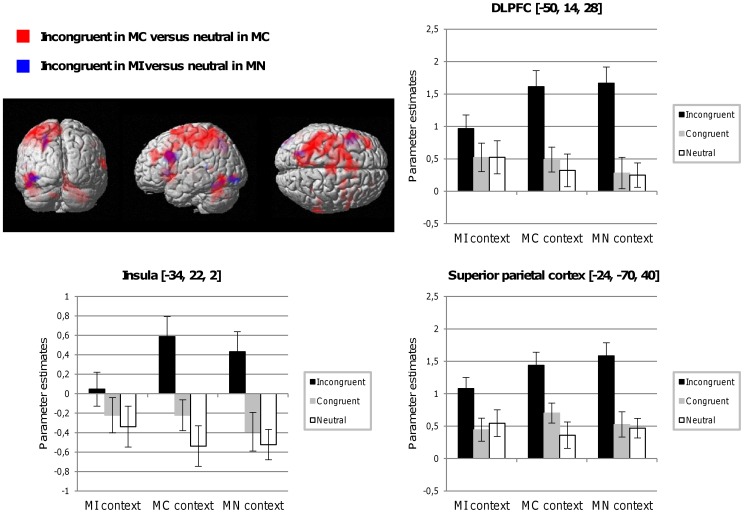
fMRI results for the conjunction analysis. Common activation in the left hemisphere between incongruent trials in the MI versus neutral trials in the MN context (blue) and incongruent trials in the MC versus neutral trials in the MC context (red) (statistical threshold at *p*<.001 uncorrected for the present display). Bar graphs illustrate the mean parameter estimates for brain areas that emerged in the conjunction analysis (DLPFC, insula, and superior parietal cortex), and are displayed for the different item types (incongruent, congruent, and neutral) in the three contexts. Error bars represent standard errors.

A similar repeated measures ANOVA was conducted for the accuracy data (percentage of errors and no response) for the three types of items in the three contexts (Table 1). This analysis only revealed a main effect of item type (*F*(2,48) = 25.27; *p*<.0001; 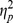 = .51), showing more errors for incongruent trials, independently of the context. We failed to show any main effect of context, although it was very close to significance (*F*(2,48) = 3.13; *p* = .05; 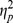 = .12), or any context × item type interaction (*F*(4,96) = 1.20; *p* = .31; 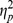 = .05).

**Table 5:** Common and specific activations between (incongruent trials of MC – neutral trials of MC) and (incongruent trials of MI – neutral trials of MN) as revealed by conjunction and interaction analyses.

Hemisphere	Anatomical region	MNI coordinates			Z score	P value (uncorrected)
		x	y	z		
**a) Common activations – conjunction analysis**						
L	Superior parietal	−24	−70	40	4.19	**<.001**
L	Inferior frontal	−50	14	28	4.01	**<.001**
L	Inferior frontal	−56	18	36	3.65	**<.001**
L	Anterior insula	−34	22	2	3.87	**<.001**
L	Fusiform gyrus	−48	−56	−20	3.78	**<.001**
L	Inferior occipital	−44	−66	−12	3.63	**<.001**
L	Inferior occipital	−44	−86	−4	3.46	**<.001**
**b) Specific activations – interaction analysis**						
L	Middle frontal	−28	−12	54	4.85	**<.001**
L	Middle frontal	−48	−16	60	4.40	**<.001**
L	Middle frontal	−36	−20	70	4.28	**<.001**
R	Middle frontal	32	−8	56	3.40	**<.001**
R	Superior frontal	16	50	18	4.16	**<.001**
R	Superior frontal	12	26	58	3.58	**<.001**
R	Superior frontal	6	48	42	3.49	**<.001**
R	Superior frontal	8	40	44	3.33	**<.001**
R	Inferior frontal	44	12	16	3.61	**<.001**
L	Anterior cingulate	−6	20	46	3.37	**<.001**
L	Anterior insula	−32	16	−8	3.58	**<.001**
R	Cerebellum	10	−56	−12	4.55	**<.001**
R	Cerebellum	6	−62	−8	4.37	**<.001**
R	Cerebellum	22	−50	−24	3.93	**<.001**
L	Cerebellum	−32	−48	−50	4.12	**<.001**
L	Cerebellum	−14	−68	−32	3.57	**<.001**
L	Cerebellum	−10	−40	−50	3.55	**<.001**
L	Cerebellum	−32	−70	−24	3.31	**<.001**
R	Superior temporal	68	−44	10	3.75	**<.001**
L	Inferior temporal	−46	−66	−2	3.35	**<.001**
L	Midbrain (colliculus)	−8	−26	−10	4.00	**<.001**
L	Thalamus	−14	−30	−4	3.88	**<.001**
L	Thalamus	−18	−24	8	3.80	**<.001**
R	Caudate nucleus	12	8	2	3.73	**<.001**

L/R = left or right; x, y, z: coordinates (mm) in the stereotactic space defined by the Montreal Neurological Institute (MNI). Both conjunction and interaction analyses were conducted with a *p* value <.001 uncorrected.

### fMRI Data

#### General interference effect

First of all, the general interference effect (i.e., incongruent vs. neutral items) across the three contexts revealed a large map of activation corresponding to the extensive fronto-parietal network typically associated with interference resolution in the Stroop task (see Table 2). More specifically, we found strong activation in ACC, DLPFC, inferior and superior parietal regions, and also the insula and cerebellum when interfering items were presented.

#### Transient patterns of brain activation

We first analyzed neural correlates of the interference effect for the three contexts separately (incongruent vs. neutral). As expected, the interference effect in the MC context was associated with strong differential activation between incongruent and neutral trials, especially in fronto-parietal areas, including the DLPFC and ACC (see Figure 3, Table 3a). The interference effect in the MI context showed no differential activation between incongruent and neutral items in this context (Table 3b). The interference effect in the MN context elicited activations in the superior parietal lobe, DLPFC, and insula (Table 3c).

In addition, we also contrasted incongruent trials in the MC and MI contexts with neutral trials in the MN context. Using these items as a baseline for comparison is useful, since they are processed independently of congruency expectations (due to the MN context) that would modulate the involvement of word-reading processes. These results did not differ from the previous contrast for the MC context and confirmed the involvement of the fronto-parietal network including the DLPFC and ACC (Table 4a). In addition, they showed an increased activation in the DLPFC for incongruent trials in the MI context (Table 4b), in a similar area to that found for incongruent trials in the MC and MN contexts.

To further investigate similarities in the activation maps for incongruent trials in the MI and MC contexts, we conducted a conjunction analysis between contrasts assessing the interference effect in the MI context (incongruent items in the MI context vs. neutral items in the MN context) and the interference effect in the MC context (incongruent vs. neutral items in the MC context). As shown in Figure 4 and Table 5a, incongruent trials in both contexts elicit, to some extent, activations in a similar brain network including the DLPFC, superior parietal cortex, and insula. However, this conjunction analysis did not show common activation in various frontal, cingulate, and cerebellar regions, which therefore seem to be specific to incongruent trials in the MC context. This was confirmed by an interaction analysis between the same contrasts ((incongruent items in MC - neutral items in MC) vs. (incongruent items in MI - neutral items in MN)), which showed that the superior and middle frontal gyrus, anterior cingulate, and cerebellum (Table 5b) were specifically activated for incongruent trials in the MC context.

#### Sustained patterns of brain activation

The main evidence of proactive control we expected to see was sustained (across trials) increased activation in the lateral PFC in the MI context, but not in the MC and MN contexts. Hence, in further analyses, we directly contrasted the MI context with the MC and MN contexts. More specifically, brain activity associated with the processing of the three kinds of items in the MI context was contrasted with brain activity associated with the processing of these items, first in the MC context, and next in the MN context. The results did not confirm our hypothesis. Indeed, no matter which contrast was considered (MI vs. MC or MI vs. MN), no significant difference in brain activation emerged.

## Discussion

Using a variant of the Stroop task composed of three different contexts (mostly congruent, mostly incongruent, and neutral), this study investigated the proposition derived from the Dual Mechanisms of Control account [11], [12] that participants would adopt a reactive control strategy in mostly congruent condition, and a proactive control strategy in mostly incongruent condition.

At a behavioral level, our findings globally supported our predictions when considering reaction times data. Indeed, we replicated the well-known proportion congruent effect [13]–[15]. More specifically, whereas interference from incongruent trials was present in all three contexts, reaction times were slower for incongruent trials in the MC than in the MI context, which is in agreement with the hypothesis that participants adopt a reactive control strategy. Given the rarity of incongruent trials in this context, they would rely on word-reading processes, since this strategy gives rise to the fastest and most correct responses for the majority of trials. However, when interference occurred, the experienced conflict was even greater since it was unexpected, in comparison to incongruent trials in the MI context, in which participants are assumed to focus strongly on task-relevant information (i.e., not reading the words but focusing on color naming) given the high expectation of conflict. In addition, we observed a facilitation effect in the MC but not in the MI context, which is also in agreement with our hypothesis of a smaller facilitation effect in MI context due to lower reliance on word reading. However, one must be cautious in the interpretation of the facilitation effect. Indeed, the comparison of performance in the MI and MC contexts with performance in the MN context brought some surprising findings. First, a larger interference effect was not observed in the MC by comparison to the MN context (slower reaction times for incongruent trials in the MN context). Second, the facilitation effect was not reduced in the MI context (the facilitation effect was absent in both the MI and MN contexts). A characteristic of our experimental design that may have contributed to these unexpected findings is the use of a row of X’s as baseline. More specifically, it has been argued [9] that the use of non-words, such as X’s as baseline, creates a confound between lexicality and congruency (neutrals items differ both in terms of lexicality and congruency from congruent and incongruent items, these ones differing only in terms of congruency). In that line, Brown [9] showed that reaction times were faster for X’s compared to neutral words, creating then smaller facilitation and larger interference effects when a row of X’s was used as baseline. Therefore, it might be interesting to investigate proactive and reactive control distinction within a proportion congruency manipulation that controls for this confound. Finally, contrary to our expectations, error data analysis did not show any modulation of error rates according to the context. We only observed a globally higher rate for incongruent trials compared to congruent and neutral trials. However, accuracy is usually much less sensitive than reaction times, since error rates are typically very low, especially for congruent and neutral trials, and not consistently influenced by proportion congruency [13], [14], [16] **.** This low sensitivity could explain the absence of congruency effect observed here.

Regarding brain imaging data, the results did not perfectly match our predictions of a transient reactive mode for the MC context versus a sustained proactive mode for the MI context. For reactive control, our analyses supported the postulated neural network and showed that incongruent trials in the MC context (contrasted with neutral trials in the MC or MN context) were associated with a large fronto-parietal pattern of activation, including the ACC and DLPFC, the two core brain areas postulated to underlie reactive control, but also the inferior and superior parietal cortex and anterior insula, which constitute brain areas frequently reported to be active during conflict processing in the Stroop literature (for a review, see [21], [22]) and in cognitive control studies in general. The ACC is typically conceived of as responsible for detecting conflict between incompatible response tendencies, whereas the subsequent joint involvement of the DLPFC and parietal cortex is responsible for implementing strategic adjustments in top-down control in order to select the appropriate response (through activation of task-relevant information [1], [39]). Interestingly, it has been proposed that the role of parietal cortex may be to represent the different response possibilities [40], permitting the DLPFC to focus on task-relevant information and select the corresponding appropriate response. As for the anterior insula, this brain area has repeatedly been associated with a multitude of cognitive tasks (for a review, see [41]), and is hypothesized to play a major role in identifying salient stimuli (important environmental stimuli) within the stream of items [41], [42], such as incongruent trials in our MC context. It would then facilitate the processing of task-relevant information through transient control signals before the subsequent involvement of the fronto-parietal network, which is responsible for control implementation.

fMRI analyses for proactive control failed to evidence any increase in sustained activity of the lateral PFC in the MI context. With reference to the works of Braver and colleagues, one important consideration has to be taken into account. Indeed, most of their studies investigated proactive control using experimental tasks such as the AX-CPT task [28], [29], task-switching paradigms [31], or working memory tasks [30], which used contextual cues as task goal-relevant information regarding the correct response to make to the following probes. This kind of procedure could favor the use of a proactive control strategy, given that the delay between the cue and the probe constitutes a period during which relevant information is actively maintained in order to successfully accomplish the task. In this regard, it could be useful to investigate the proportion congruent effect in the Stroop task by providing a cue before each item that would influence the extent to which participants focus on task-relevant information (i.e., color naming rather than word reading). In that line, Braver [12] recently emphasized the importance of strong and reliable contextual cues in the implementation of a proactive strategy.

In addition, event-related analysis regarding incongruent trials in the MI context also produced some surprising results. First, we did not see evidence of any differential pattern of activation between incongruent and neutral trials within the MI context, in agreement with one postulated property of proactive control over similar processing for all items belonging to the MI context (i.e., similar inhibition of word reading and task-relevant focus). Second, we found transient increased activation in the DLPFC when incongruent trials in the MI context were contrasted with neutral trials in the MN context. One might wonder why this comparison evoked DLPFC activation. Indeed, neutral items in both MI and MN contexts were not expected to differ in terms of the involvement of word reading. However, the reason for this suppression of word reading was not the same in the two task contexts (i.e., dampening of word-reading processes due to high expectation of interference in MI, but due to the uninformative nature of the word dimension in the MN context where the majority of trials were non-words). Therefore the observed DLPFC activation could reflect the task-relevant focus (i.e., not reading words) at play when interference occurs in the MI context, which cannot be observed in the within-context contrast because of the similar processing strategy applied to all items. Moreover, conjunction analysis showed a common involvement of this DLPFC area but also of the superior parietal cortex and insula for incongruent trials in both MC and MI contexts. This finding confirmed the conflict sensitivity of this fronto-parietal network, which was involved every time incongruent trials were encountered.

However, other components of this conflict resolution network were only sensitive to the degree of experienced conflict as shown by the interaction analysis, which found activation in the ACC, superior, middle, and inferior frontal areas, and cerebellum for rare conflicting events only (i.e., incongruent trials in the MC context). This finding is in agreement with the work of Carter et al. [24], which found the ACC to be sensitive to the global amount of conflict within a block or list of stimuli, and supports the evaluative role of the ACC in conflict detection, as well as the subsequent involvement of the DLPFC in conflict resolution through cognitive control implementation [26], [43]. Strong reliance on word reading in the MC context would cause ACC activation, whereas strong task-relevant focus in the MI context would “skip” this step in conflict processing (i.e., no ACC involvement) and directly engage top-down areas (e.g., DLPFC) to continue to overcome the tendency to read words. Regarding the anterior insula, we found activation of this structure in both the conjunction and interaction analysis, which can be explained by reference to this structure’s sensitivity to salient environmental stimuli. Indeed, incongruent trials can be considered as salient events in both MC and MI contexts (insula activation in the conjunction analysis), but especially salient in the MC context given their unexpected nature (insula activation in the interaction analysis).

Overall, behavioral and brain findings of the present experiment did not totally match with our predictions. Indeed, whereas behavioral data replicated the proportion congruent effect and were in agreement with the proactive/reactive distinction, fMRI data did not support the sustained nature of the proactive control mechanism. However, we nevertheless consider that two different modes of control were effectively at play in MC and MI contexts. Indeed, in the MI context, the smaller interference effect on reaction times and the absence of ACC involvement for incongruent trials indicates that the level of experienced conflict was reduced. This reduction could originate from a higher relevant task focus in that context (low reliance on word reading processes). However, contrary to the proactive mechanism postulated within the DMC account [11], the strong goal relevant focus at play here was mainly a transient process, as attested by the absence of sustained increased activation, and the transient DLPFC activation for incongruent trials. In fact, this transient activation and the smaller interference effect at the behavioral level could indicate item-specific control. More specifically, a proactive strategy (in the sense of low reliance on word reading and strong task goal focus) could be engaged at stimulus onset in the MI context and for incongruent items only, rather than before the item presentation and in a sustained manner as postulated by Braver et al. [11].

In this context of an item-specific control mechanism, some authors have recently questioned the validity of accounting for the proportion congruent effect in terms of variations in control strategy at the global (i.e., list-wide) level [44]–[46] and its proposal that the involvement of word-reading processes is modulated as a function of general task context and the associated expectations [15], [18]. More specifically, they showed that the proportion congruency effect might be accounted for by control mechanisms that operate at the item level, at the time of stimulus onset (stimulus-driven). These item-specific mechanisms are said to be implemented transiently on a trial-by-trial basis in response to the information associated with each particular item, rather than with the list. In this view, the proactive control mechanism, in the sense of sustained preparatory attention prior to the occurrence of the next item, is difficult to defend. However, other recent works by Bugg and colleagues [47], [48] have provided evidence against a pure item-specific list-wide proportion congruent effect, by clearly demonstrating the involvement of a list-level control mechanism minimizing the influence of word-reading processes when item-specific influences were controlled for (see also Hutchison [49], for a demonstration of a list-wide congruency effect not confounded with item-specific effects). Importantly, these authors interpreted their behavioral findings by referring to the proactive nature of these list-wide control mechanisms.

In this context, the present experiment does not allow us to decide about the responsible mechanism at play during the proportion congruent effect observed. Indeed, even if the involvement of item-specific mechanism is a possibility, our study was not explicitly designed to separate and evaluate the respective influences of list-wide and item-specific mechanisms. Indeed, list-wide proportion congruency was equivalent for each word of the stimulus set (e.g., in the MC context, the four color words were presented equally often in a congruent way). Finally, the absence of differential activation between incongruent and neutral items in the MI context, which argued in favor of similar processing for all items in this context, is more compatible with list-level control rather than an item-specific mechanism. Therefore, further fMRI studies specifically addressing this issue are needed in order to respond to the question raised by our study regarding the temporal dynamics of the proactive control mechanism when investigated with a list-wide proportion congruency manipulation. In addition, as mentioned above, the use of non-words as baseline in the present study might have influenced the magnitude of our interference and facilitation effects, and thus the engagement of the associated brain areas.

In conclusion, both behavioral and brain imaging results of the present experiment confirmed the involvement of two distinct control strategies according to the task context (i.e., proportion congruency in a series of trials). However, the brain findings raised questions about the involvement of a proactive mechanism, defined as a sustained mechanism throughout a block of stimuli. Along these lines, we stressed the importance of experimental design and procedure characteristics that could explain the lack of sustained activation. In addition, we raised the possibility that high task-relevant focus, one core property of proactive control, could originate from item-specific rather than list-wide mechanisms.
